# *Withania* *somnifera* Root Extract Inhibits Mammary Cancer Metastasis and Epithelial to Mesenchymal Transition

**DOI:** 10.1371/journal.pone.0075069

**Published:** 2013-09-12

**Authors:** Zhen Yang, Anapatricia Garcia, Songli Xu, Doris R. Powell, Paula M. Vertino, Shivendra Singh, Adam I. Marcus

**Affiliations:** 1 Department of Hematology and Medical Oncology, Winship Cancer Institute of Emory University, Emory University School of Medicine, Atlanta, Georgia, United States of America; 2 Yerkes National Primate Research Center, Emory University School of Medicine, Atlanta, Georgia, United States of America; 3 Department of Radiation Oncology, Winship Cancer Institute of Emory University, Emory University School of Medicine, Atlanta, Georgia, United States of America; 4 Department of Pharmacology & Chemical Biology, and University of Pittsburgh Cancer Institute, University of Pittsburgh School of Medicine, Pittsburgh, Pennsylvania, United States of America; University of Colorado, Boulder, United States of America

## Abstract

Though clinicians can predict which patients are at risk for developing metastases, traditional therapies often prove ineffective and metastatic disease is the primary cause of cancer patient death; therefore, there is a need to develop anti-metastatic therapies that can be administered over long durations to specifically inhibit the motility of cancer cells. *Withania* *somnifera* root extracts (WRE) have anti-proliferative activity and the active component, Withaferin A, inhibits the pro-metastatic protein, vimentin. Vimentin is an intermediate filament protein and is part of the epithelial to mesenchymal transition (EMT) program to promote metastasis. Here, we determined whether WRE standardized to Withaferin A (sWRE) possesses anti-metastatic activity and whether it inhibits cancer motility via inhibition of vimentin and the EMT program. Several formulations of sWRE were created to enrich for Withaferin A and a stock solution of sWRE in EtOH could recover over 90% of the Withaferin A found in the original extract powder. This sWRE formulation inhibited breast cancer cell motility and invasion at concentrations less than 1µM while having negligible cytotoxicity at this dose. sWRE treatment disrupted vimentin morphology in cell lines, confirming its vimentin inhibitory activity. To determine if sWRE inhibited EMT, TGF-β was used to induce EMT in MCF10A human mammary epithelial cells. In this case, sWRE prevented EMT induction and inhibited 3-D spheroid invasion. These studies were taken into a human xenograft and mouse mammary carcinoma model. In both models, sWRE and Withaferin A showed dose-dependent inhibition of tumor growth and metastatic lung nodule formation with minimal systemic toxicity. Taken together, these data support the hypothesis that low concentrations of sWRE inhibit cancer metastasis potentially through EMT inhibition. Moreover, these doses of sWRE have nearly no toxicity in normal mouse organs, suggesting the potential for clinical use of orally administered WRE capsules.

## Introduction

Breast cancer is one of the most common cancers among women in the United States and the second leading cause of female cancer death [1]. Greater than 90% of breast cancer patient deaths are attributed to metastatic disease where the primary tumor has invaded distant sites. Since these metastatic cells are often highly aggressive, difficult to detect, and chemoresistant [2], one therapeutic strategy could be to prevent metastatic disease rather than treat it once it occurs.

The metastatic process can be categorized into three stages- tumor cell invasion into surrounding tissue, intravasation into blood or lymphatic vessels, and extravasation into a new host environment [3-5]. In many cases, metastatic cells undergo epithelial-to-mesenchymal transition (EMT), where genetic and epigenetic events cause a polarized epithelial cell to become migratory and invasive [6]. At the molecular level, EMT causes a gain of vimentin and fibronectin expression, and loss of E-cadherin at the cell membrane [7,8]. Accumulating evidence shows that EMT is a major mechanism driving breast cancer progression and metastasis [9-13].

*Withania* *somnifera* (Ashwagandha) plants are widely used in East Indian medicine. The W. somnifera root extract (WRE) is composed of 14 compounds known as withanolides, with Withaferin A being the most prominent [14-16]. Withaferin A is a steroidal lactone that induces apoptosis and inhibits tumor growth by targeting signaling proteins such as P53, FOXO3A, Notch-1, Hsp90 and STAT3 [17-22]. Withaferin A treatment leads to cell cycle arrest, increased reactive oxidative stress [23-26], inhibition of the JAK/STAT pathway [27], inhibition of angiogenesis [28], and modified cell shape and behavior [29]. Withaferin A also possesses potent anti-invasive activity, which could potentially be due to its interaction with the pro-migratory protein vimentin.

Vimentin is type III intermediate filament and classical EMT protein marker [30]. Though vimentin functions in maintaining cell structure, it is also a highly dynamic polymer that assembles and dissembles in a motile cell. When cells undergo EMT, vimentin expression is increased and this is thought to provide cells with a more mesenchymal, pro-motile phenotype [31]. The precise role of vimentin during EMT and development is unclear, since a vimentin mouse knockout model did not show severe developmental defects [32]. Additional studies however did go on to show that these mice had impaired fibroblast wound healing, and a reduced capacity to contract a collagen network [33].

Withaferin A is proposed to bind to vimentin through a covalent modification of cysteine 328 [34], leading to changes in vimentin morphology and phosphorylation [29]; however, other data show that mutation of cysteine 328 does not impact Withaferin A-induced vimentin inhibition [29]. When given intra-peritoneally (IP), Withaferin A effectively inhibits breast cancer metastasis and has nearly no observable toxicity [35].

Less is known about the anti-tumor activity of the Withaferin A parent root extract, WRE. Similar effects are observed on tumor growth, cell cycle, and angiogenesis with WRE treatment [36-40] along with immunomodulatory effects in colon and lung cancer [41,42]. Nevertheless, studies directly comparing WRE to Withaferin A have not been performed, and its anti-metastatic activity has not been well studied. Furthermore, WRE possesses several advantages over Withaferin A, since it can be given orally in a capsule and the active withanolides could have pharmacological synergy; therefore, we wanted to determine the anti-metastatic efficacy of WRE standardized (sWRE) to the pure active component, Withaferin A, in breast cancer. We showed that sWRE can inhibit human breast cancer cell invasion *in vitro* and metastasis in both allograft and xenograft breast cancer mouse models, similar to the pure small molecule Withaferin A. sWRE induces vimentin reorganization and morphologic cellular changes in human breast cancer cells and, importantly, inhibits EMT induction in normal human mammary epithelial cells. Our results shed light on the potential of sWRE as a novel complementary alternative medicine used as an anti-metastatic preventative therapy in high-risk breast cancer patients.

## Methods

### Reagents and antibodies

WFA was purchased from Chromadex (Irvine, CA) and WRE was provided by Verdure Sciences (Noblesville, IN) with the certificate of analysis stating that it is free of heavy metals, bacteria, and fungus. The antibody against vimentin was purchased from Sigma (St. Louis, MO), E-cadherin from BD Biosciences (Bedford, MA), fibronectin from Abcam (Cambridge, MA), and GAPDH from Cell Signaling (Beverly, MA).

### sWRE preparation

100% ethanol was heated to 60°C and then mixed with WRE to the concentration of 250mg/ml for 30 minutes in a glass beaker. Distilled H_2_O was then slowly added to lower the ethanol concentration to 90%, and stirring continued for another 30 minutes. The mixture was then spun at 4000rpm in a centrifuge for 15 minutes at room temperature and the supernatant was collected. The supernatant was then passed a 0.22µm filter, aliquoted, and kept at -80°C for future use.

### Cell lines and culture conditions

Human MDA-MB-231 (ATCC # HTB-26), MCF-7 (# HTB-22) and T47D (# HTB-133) breast cancer cell lines were purchased from the American Type Culture Collection (ATCC, Manassas, VA). Hs578-T, HCC1806 and MDA-MB-468 human breast cancer cell lines were provided by Dr. O, Regan (Emory University [45]). Human MCF10A mammary epithelial cell line was provided by Dr. Vertino (Emory University [45]). Murine breast carcinoma 4T1 cells were provided by Dr. Dewhirst (Duke University [35,45]). T47D and HCC1806 were grown in RPMI 1640 with 10% FBS. MDA-MB-231, MCF-7, Hs578-T, MDA-MB-468 and 4T1 were grown in DMEM 10% FBS. MCF10A cells were grown in DMEM/F12 supplemented with 5% FBS, 20ng/ml EGF, 0.5µg/ml Hydrocortisone, 100ng/ml cholera toxin, and 10µg/ml insulin. All cell lines were maintained in a humidified incubator at 37°C in a 5% CO_2_ atmosphere.

### Cytotoxicity assay

The Promega CellTiter 96^®^ AQueous Non-Radioactive Cell Proliferation Assay (MTS) was performed for determining the *in vitro* cytotoxicity of sWRE. Briefly, cells were cultured in 96-well plates overnight and then treated with sWRE at the indicated concentration for 72 hours. Cell viability was assessed by determining the absorbance at 490nm as described by the manufacturer (Promega, Madison, WI). Cell viability was expressed as: A_exp group_ /A_control_ X 100.

### In vitro wound healing assay

A cell migration wounding assay was performed as described previously [43]. Cells were cultured in 6 wells plate to 100% confluency. After wounding with a pipette tip, cells were washed with PBS and new media with the respective concentration of sWRE was added. Cells then were allowed to migrate for 24 hours at 37°C. Images of cells were taken at time 0 and 24 hours with an Olympus IX51 widefield microscope (Center Valley, PA) at 5X magnification with a Hamamatsu Orca ER CCD camera.

### Matrigel invasion assay

Cell invasion was assayed using the Roche xCelligence RTCA DP (Real-Time Cell Analyzer Dual Plate) Instrument (Indianapolis, IN). DMEM with 10% FBS was added into the bottom chamber of a CIM-Plate 16. 250µg/milliliter Matrigel (BD Biosciences) was polymerized in the wells of the top chamber for 1 hour at 37°C. Serum free media was added to the top chamber, incubated for 30 minutes at 37°C, and a background measurement was taken. Cells were pretreated with different concentrations of sWRE in serum free media over night. These cells were then added to the top chamber and the plate was incubated in 37°C with the xCelligence plate reader. Impedance measurements were taken in the bottom well and the impedance increase correlates to increasing numbers of migrated cells. Changes in impedance, which is reflected by the cell invasion index values, were monitored for at least 24 hours.

### Western blotting

Protein levels in cell lysates were measure using a standard BCA assay. Cell lysates in sample buffer were separated on a 10% SDS-PAGE gels, and were transferred to PVDF membranes (Bio-Rad, Hercules, CA). After blocking with 5% fat-free milk-containing TBST, the membranes were separately incubated with antibodies to vimentin, E-cadherin, fibronectin, and GAPDH at room temperature for 2 hours. Membranes were then washed and incubated with horseradish peroxidase conjugated secondary antibodies for 1 hour. The bands were detected using Amersham ECL Plus reagents and then exposed to film.

### Confocal imaging

Cells were cultured on glass cover slips and treated with sWRE for 24 hours. Cells were then fixed and processed for immunofluorescence microscopy as previously described [35]. Cells were stained using a primary anti-vimentin antibody and secondary Alexa 488-conjugated goat anti-mouse IgG. Coverslips were mounted onto slides and imaged using Zeiss LSM510 META confocal microscope with a 40X Plan-NEOFLUAR oil objective (NA 1.3).

### Real-time PCR

Total RNA was isolated from cells using the RNeasy Mini kit (Qiagen, Valencia, CA) and pretreated with DNase I. cDNA was then synthesized using random hexamer primers and MMLV- reverse transcriptase. Target-specific primers were used to amplify cDNA in triplicate using a reaction mixture that contained 1µl of the appropriately diluted cDNA sample, 0.2µmol/l primers and 12.5µl of IQ SYBR Green supermix (Bio-Rad). 18S rRNA was amplified from the same samples as an internal control. The reaction was subjected to a hot start for 3 min at 95°C and 40 cycles of 95°C, 10 s; 55°C (18S) or 63°C (vimentin), 30 sec. Primers for real-time PCR analysis were for vimentin: 5’ AATGGCTCGTCACCTTCGTGAA3’ and 5’ CAGATTAGTTTCCCTCAGGTTCAG3’ and for 18S, 5’ GAGGGAGCCTGAGAAACG G3’ and 5’ GTCGGGAGTGGGTAATTT GC3’

### Three dimensional spheroid invasion assay

Agarose-coated plates were made by loading each well with 0.75% agarose in DMEM with 10% FBS. After gelation, MCF10A cells were added into the wells and incubated at 37°C. Cell spheroids formed within 3-5 days. 5-10 spheroids were mixed with Matrigel in DMEM or DMEM plus sWRE. The mixture was placed in the middle of a 35 mm imaging petri dish with a #1.5 coverslip (MatTek Corporation) and then sandwiched on top with an additional cover slip. The dish was then placed in a 37 °C incubator for 30 min to allow for Matrigel polymerization, then 2ml of DMEM with 10% FBS was added. The dish was transferred to the live cell PerkinElmer Ultraview ERS spinning disk confocal microscope [44] and images were acquired using 20X Zeiss objective (NA 0.75) every 20 minutes for 24 hours with a Hamamatsu Orca ER camera.

### In vivo sWRE toxicity study

Eight week old female Balb/c mice were purchased from Harlan and housed in the Winship Cancer Animal Models facility according to our approved IACUC protocol. Mice were kept in groups of five per cage and fed with AIN76A control diet and water *ad libitum*. The mice were randomized into 3 groups of 3 mice per group and treated by oral gavage with either vehicle (9% ethanol) or vehicle containing sWRE at 4, and 8mg/kg body weight, 3 times a week for 4 weeks. Mice body weight was recorded each time after oral gavage. After 4 weeks of treatment, mice were sacrificed and organs (heart, lung, liver, spleen and kidney) were collected and sent for pathological analysis. Toxic effects were evaluated by a blinded veterinarian pathologist based upon the presence of inflammation, necrosis and fibrosis using a scale from normal (1+) to moderate (3+).

### Mammary carcinoma model

All mouse studies were performed in accordance with our approved Emory University Institutional Animal Care and Use Committee protocol. Eight weeks old female Balb/c mice were divided randomly into 4 groups with 10 mice in each group. 10^6^ 4T1 cells in PBS were injected subcutaneously into the mammary fat pad of each mouse. One week after injection, sWRE were given by oral gavage with either vehicle or vehicle containing sWRE at 1, 4, and 8mg/kg body weight 3 times a week for 4 weeks. Withaferin A was intraperitoneally injected by dissolving in 10% DMSO, 20% Cremophor-EL and 50% PBS at 1, 4, and 8mg/kg 3 times a week for 4 weeks. The tumor volume was recorded before gavage using calipers with volume=(width)^2^ x length/2. Mice were sacrificed after 4 weeks of treatment and metastatic lung nodules were counted under a dissecting microscope. For H&E staining, the lung and primary tumor from vehicle and sWRE-treated mice were fixed in 10% neutral-buffered formalin, processed, embedded in paraffin, and sectioned at 5µm thickness. Representative tumor sections from vehicle control, sWRE-treated, and Withaferin A-treated mice were processed for H&E staining.

### Xenograft mouse breast cancer model

All mouse studies were performed in accordance with our approved Emory University Institutional Animal Care and Use Committee protocol. Eight week old female athymic nude Foxn1nu mouse were purchased from Harlan and housed in the Winship Cancer Animal Models facility. Mice were kept in groups of five per cage and fed with AIN76A control diet and water ad libitum. Mice were divided randomized into 7 groups with 10 mice in each group and 10^6^ MDA-MB-231 cells in PBS were injected subcutaneously into the mammary fat pad of each mouse. One week after injection, mice were treated with vehicle (9% ethanol), vehicle containing sWRE at 1, 4, and 8mg/kg by oral gavage, or intraperitoneally injected with Withaferin A dissolved in 10% DMSO, 20% Cremophor-EL and 50% PBS at 1, 4, and 8mg/kg 3 times a week for 4 weeks. The tumor volume was recorded before gavage. Mice were sacrificed after 4 weeks of treatment and metastatic lung nodules were counted under a dissecting microscope. For H&E staining, the lung and tumor from vehicle and sWRE-treated mice were fixed in 10% neutral-buffered formalin, processed, embedded in paraffin and sectioned at 5µm thickness. Representative tumor sections from control and sWRE-treated mice were processed for H&E staining.

### Statistical Analysis

Data were analyzed statistically using GraphPad Prism for Windows (version 5). One-way ANOVA was carried out to compare the mean of lung nodules among the experimental groups. Linear mixed effects model was used to compare the mean tumor volumes among the groups. Values of p<0.05 were considered significant.

## Results

### WRE Standardization, Solubility, and Cytotoxicity

**Figure 1:**
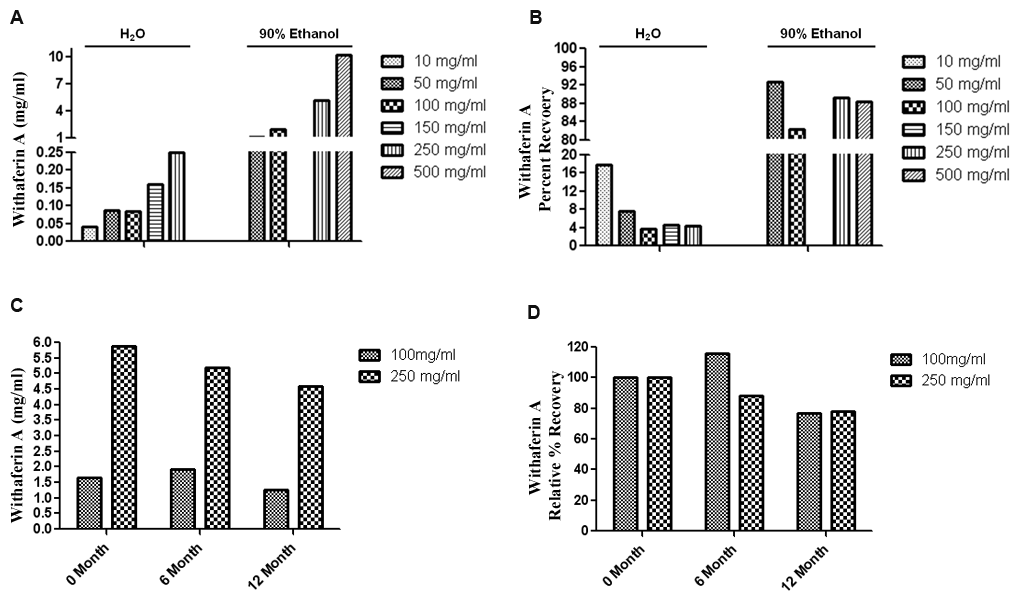
Withaferin A recovery in sWRE. sWRE powder was dissolved in different solvents and the concentration (A) and recovery rate (B) of Withaferin A in sWRE was measured by HPLC. The stability of Withaferin A in sWRE (90% EtOH stock solution) over time is shown in a bar graph with the absolute (C) and relative (D) Withaferin A concentration.

WRE powder was solubilized in H_2_O or 90% ethanol (EtOH) at different concentrations to determine optimal conditions to standardize WRE (sWRE) to the pure small molecule Withaferin A. The concentration of Withaferin A in each sWRE formulation was measured by HPLC (Figure 1A) and the recovery rate of Withaferin A after solubulization was calculated for each sample (Figure 1B). The content of Withaferin A compared to other withanolides using HPLC is shown in Table 1. When sWRE at 250mg/ml was dissolved in water, the Withaferin A recovery rate was 4%. The largest percent recovery of Withaferin A in water was 16%, observed at a starting concentration of 10 mg/ml sWRE. In contrast, when WRE powder was dissolved in 90% EtOH the Withaferin A recovery rate ranged from 80-92%. These results show that re-solubilization of sWRE in 90% EtOH is clearly superior to that in H_2_O. To estimate the long-term stability of the WRE stock solution in 90% EtOH, HPLC analysis was done on WRE after 6 and 12 months of storage at -80°C. The results show that about 90% of the initial Withaferin A can be detected after 6 months, and about 80% after 12 months (Figure 1C, D).

**Table 1:** Withanolides in WRE by HPLC.

**Withanolides**	**Concentration (mg/ml**)	**Percentage (%**)
Withaferin A	5.88	79.03
Withanoside V	0.356	4.78
12-Deoxywithastramonolide	1.06	14.25
Withanolide A	0.136	1.83
Withanolne	0.0137	0.18
**Total Withanolides**	**7.44**	**100.00**

**Figure 2:**
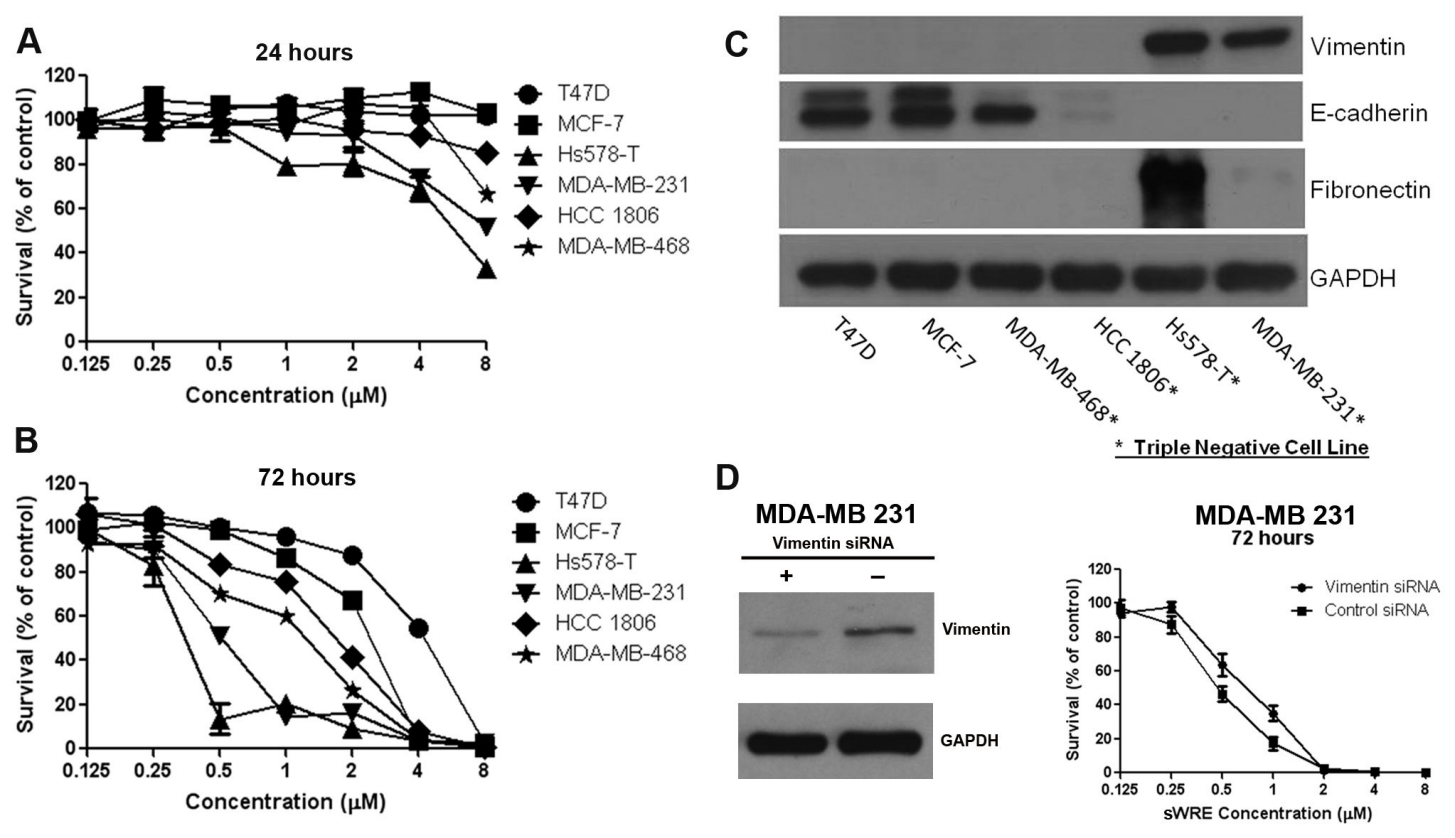
sWRE cytotoxicity in breast cancer cell lines. (A and B) Line graphs showing the % survival of six breast cancer cell lines treated with increasing concentrations of sWRE for 24 (A) and 72 (B) hours. (C) Western blot showing EMT markers in different breast cancer lines. Triple negative breast cancer cell lines are indicated with an asterisk. (D) (left) Western blot showing successful vimentin siRNA depletion (right) Cytotoxciity assay using sWRE in isogenic control and vimentin siRNA depleted cells.

To assess cytotoxicity, breast cancer cell lines were treated with increasing concentrations of Withaferin A-standardized WRE (sWRE) for 24 and 72 hours (Figure 2A, B). Among the six cell lines tested, four (MDA-MB-468, HCC 1806, Hs587-T, and MDA-MB-231) are triple negative breast cancer cell lines [45]. Since Withaferin A is a vimentin-targeting agent [30,34], vimentin expression along with other EMT markers were assessed in the cell lines. Two of the four triple negative cell lines, (Hs578-T and MDA-MB-231) were vimentin-positive and all other cell lines were vimentin-negative. These vimentin positive cell lines also exhibited EMT induction since they displayed E-cadherin loss and were fibronectin positive (Figure 2C). Interestingly, different sensitivity to sWRE was observed across the six cell lines, where the two most sensitive cell lines, Hs578-T and MDA-MB 231, were vimentin-positive with a 72 hour IC_50_ of 0.5µM and 0.4µM respectively (Figure 2B). The vimentin-negative cell lines had IC_50_s that ranged from 1.2µM to 4.0µM. To probe this, cytotoxicity assays were performed in isogenic control and vimentin siRNA depleted MDA-MB 231 cells. In this case, vimentin depleted cells show a minor decrease in sWRE cytotoxicity compared to isogenic control cells (Figure 2D).

### sWRE inhibits breast cancer cell motility and invasion, and disrupts vimentin morphology

**Figure 3:**
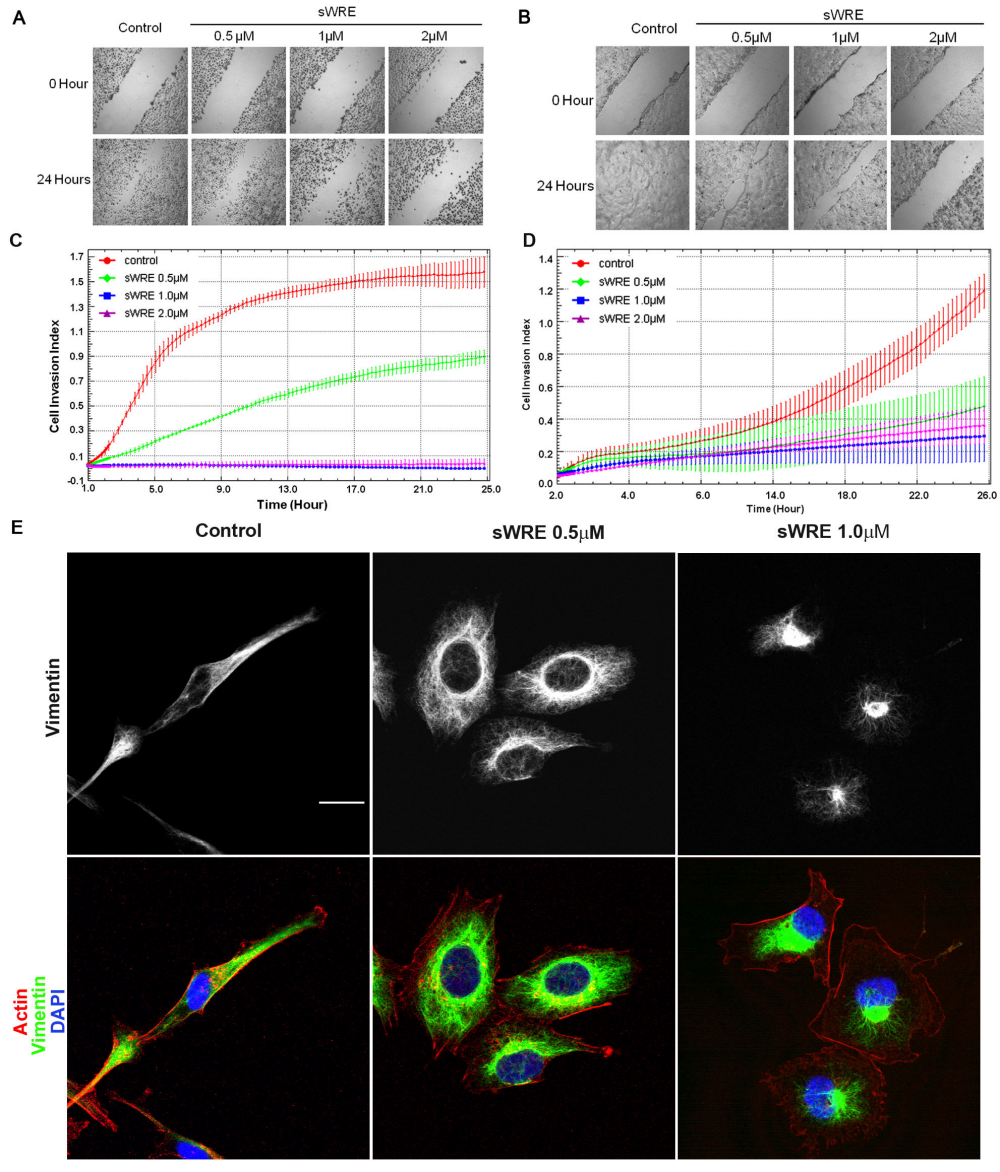
sWRE inhibits migration and invasion in MDA-MB-231 and 4T1 cell lines. (A and B) Cell wounding assay in (A) MDA-MB-231 and (B) 4T1 cells treated with increasing concentrations of sWRE for 24 hours. (C and D) Line graph showing the rate of cellular invasion through Matrigel embedded in a Boyden chamber in (C) MDA-MB-231 and (D) 4T1 cells treated with increasing concentrations of sWRE. (E) Confocal immunofluorescence imaging of vimentin (green), actin (red), and DAPI (blue) in MDA-MB-231 cells treated with 9% EtOH control, 0.5µM or 1.0µM sWRE.

We have previously shown the Withaferin A inhibits breast cancer cell motility and metastasis [35]; therefore, we sought to determine if sWRE shows anti-invasive efficacy. The effect of sWRE on the cell motility of triple negative breast cancer cells (human MDA-MB-231 and mouse 4T1) was tested using a wound healing assay. WRE inhibits cell motility in a dose-dependent manner after 24 hours of treatment at 0.5µM in both cell lines (Figure 3A,B). An expanded experiment at lower doses in MDA-MB 231 cells shows inhibition of cell motility at 0.25 µM as well (Figure S1). We then wanted to determine how sWRE impacts cell motility when vimentin protein is absent. This experiment was first attempted in MCF7 and MDA MB 468 breast carcinoma cells, both of which do not have detectable vimentin by western blotting; however in these cases, cells were also not motile so the experiment could not be performed (data not shown). Instead, we used the lung epithelial cell line, BEAS-2B, which lacks vimentin (Figure S1-B) and shows some motility. sWRE had a 24 hr anti-proliferative IC_50_ of 2.9µM and a 48 hr IC_50_ of 1.9 µM (Figure S1-C). In a wounding assay, lower doses of sWRE (0.125 to 1 µM) below the IC_50_ had nearly no impact on cell motility (Figure S1-D). It was not until treatment with 2 µM sWRE which is near the 24 hr anti-proliferative IC_50_, did we observe appreciable inhibition of motility (Figure S1-D).

The anti-invasive activity of sWRE was tested using a real time Matrigel invasion assay in a Boyden chamber. sWRE was again able to significantly inhibit cell invasion in both cell lines at doses as low as 0.25µM in 4T1 and 0.5µM in MDA-MB-231 cells (Figure 3C, D). These results show that sWRE inhibits cell motility and is anti-invasive in triple negative breast cancer cells.

Since Withaferin A inhibits vimentin [30,34], we explored whether sWRE has similar vimentin-disrupting ability. Vimentin immunofluorescence in control MDA-MB-231 cells show that vimentin is networked throughout the spindle-shaped cytoplasm; however, upon sWRE treatment (0.5µM and 1.0µM) for 16 hours, cells were not as elongated and the vimentin network was abolished (Figure 3E). Instead, vimentin accumulated as a perinuclear bundle in most cells, which is similar to the effect of pure Withaferin A [35]. Furthermore, sWRE did not decrease total cellular protein levels until 48 hrs of treatment (Figure S2), suggesting that this is not due to a defect in total protein synthesis. Therefore, based upon these observations we conclude that sWRE also possesses vimentin inhibitory activity at low doses.

### sWRE prevents TGFβ-induced EMT

**Figure 4:**
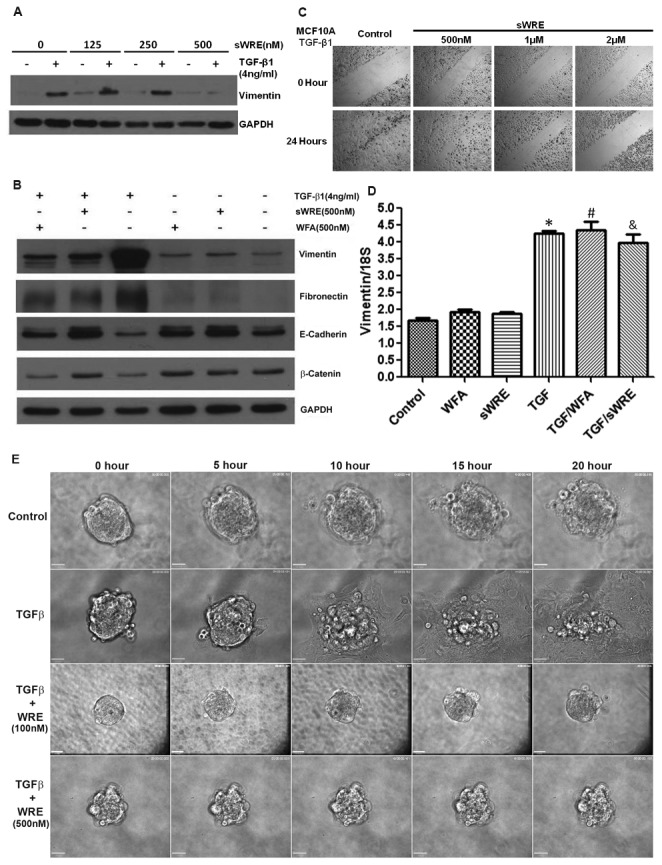
sWRE inhibits TGFβ1-induced EMT in MCF10A cells. (A) Western blot of vimentin in TGFβ1-stimulated MCF10A cells treated with increasing concentrations of sWRE. (B) Western blot of vimentin, fibronectin, and E-cadherin in TGFβ1 stimulated MCF10A cells treated with 500nM sWRE or Withaferin A. (C) Cell migration in wound-healing assay with increasing concentrations of WRE. (D) Relative vimentin mRNA levels detected by real-time PCR in TGFβ1-stimulated MCF10A cells treated with 500nM sWRE or WFA. (E) Live cell time lapse images of MCF10A 3D spheroids embedded in Matrigel and stimulated with TGFβ1. Cell were treated with 9% EtOH control, or 100nM or 500nM sWRE.

Since vimentin plays a key role in EMT, we wanted to test if sWRE could inhibit EMT and EMT-induced motility in breast cancer. To test this, we used MCF10A cells, where treatment with 4ng/ml TGFβ causes these cells to undergo EMT, as assessed by an increase in the mesenychmal markers vimentin and fibronectin, and loss of the epithelial marker, E-cadherin [8,9]. These data show that sWRE inhibits MCF10A motility in a wound healing assay with TGFβ (Figure 4C) at doses similar to those used in 4T1 and MDA-MB-231cell lines (Figure 3). To assess how sWRE impacts EMT, MCF10A cells were treated with TGFβ in the presence of sWRE or Withaferin A. TGFβ alone induced vimentin and fibronectin protein expression and decreased E-cadherin protein levels indicating successful EMT induction. In contrast, treatment with 500nM sWRE or 500nM Withaferin A potently inhibited TGFβ-induced EMT by keeping vimentin and fibronectin at pre-induction levels and increasing E-cadherin levels (Figure 4A, B). To determine if this occurs at the transcriptional level, real-time PCR of the vimentin transcript was performed and these results showed that TGFβ increased vimentin mRNA as expected, but sWRE did not impact vimentin mRNA levels (Figure 4D), similar to that observed using Withaferin A [30]. Therefore the results show that sWRE does not inhibit vimentin expression at the mRNA level but rather on the protein level.

These studies were then moved into a spheroid model of invasion to determine if sWRE inhibits EMT-induced invasion. In this model, TGFβ induced potent invasion into the Matrigel extracellular matrix using live cell imaging to visualize invasion (Figure 4E, Movie S1); however, upon treatment with doses as low as 100nM sWRE, invasion was potently inhibited (Figure 4E, Movie S2). Taken together, these results show that sWRE can inhibit EMT and EMT-induced motility and invasion.

### sWRE in vivo toxicity

**Figure 5:**
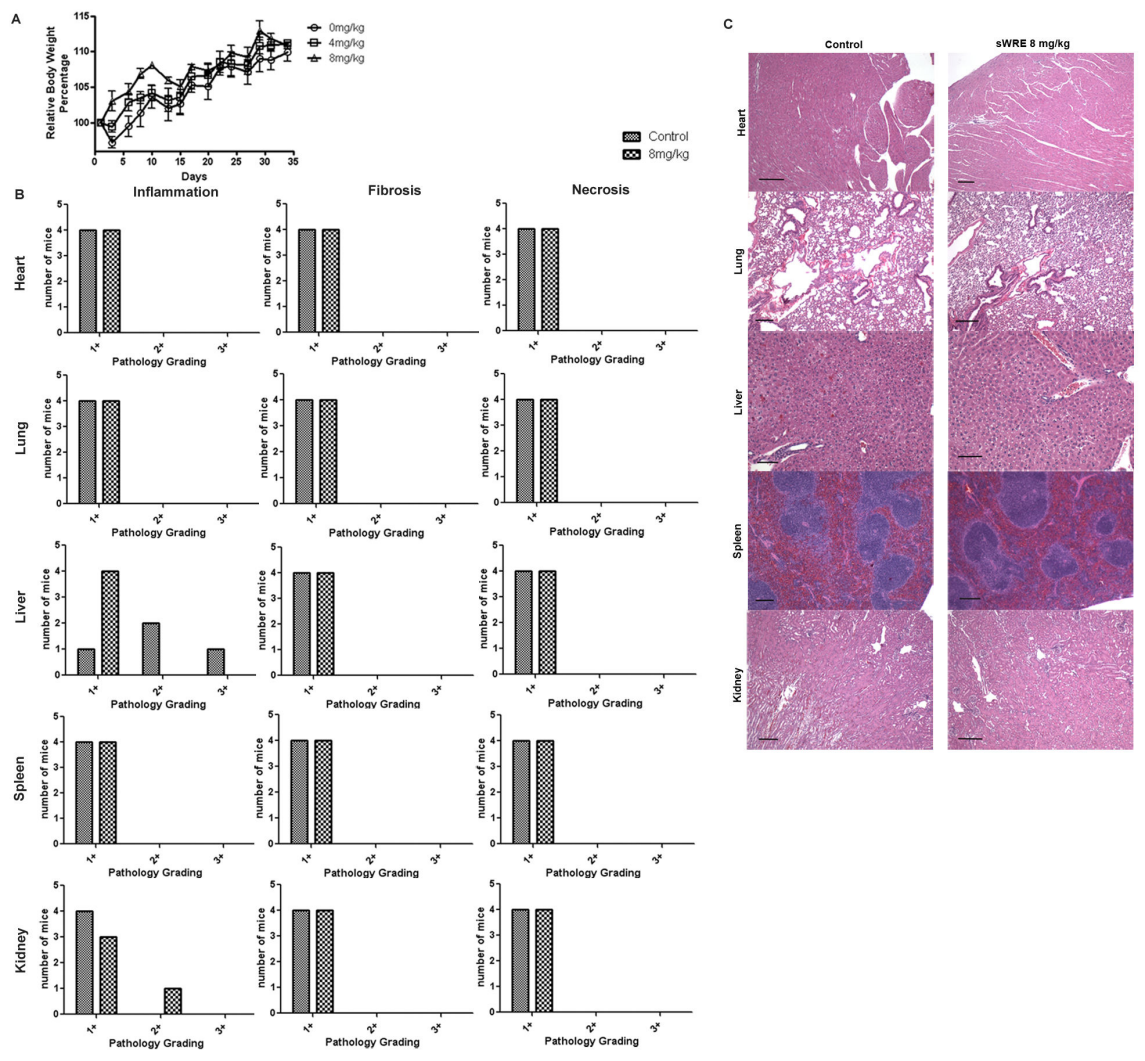
Toxicity of sWRE in female BALB/c mice. (A) Relative body weight in mice treated with vehicle, or sWRE at 4mg/kg and 8mg/kg. (B) Histological grading of inflammation, fibrosis, and necrosis in organs from mice treated with vehicle (9% EtOH) or sWRE at 8mg/kg. (C) Representative images of H&E stained histological sections.

To study the anti-metastatic efficacy of sWRE *in vivo*, sWRE toxicity was first assessed in normal female BALB/c mouse. Mice were given either 4 or 8 mg/kg sWRE in 9% EtOH and the mean body weight gain after 35 days in treated mice were not significantly different from the control group given 9% EtOH alone (Figure 5A). After four weeks of treatment, the histology of the heart, lung, liver, spleen and kidney were graded for fibrosis, necrosis and inflammation. Histological data show no significant difference between sWRE-treated and vehicle control groups (Figure 5B, C).

### sWRE anti-metastatic efficacy

**Figure 6:**
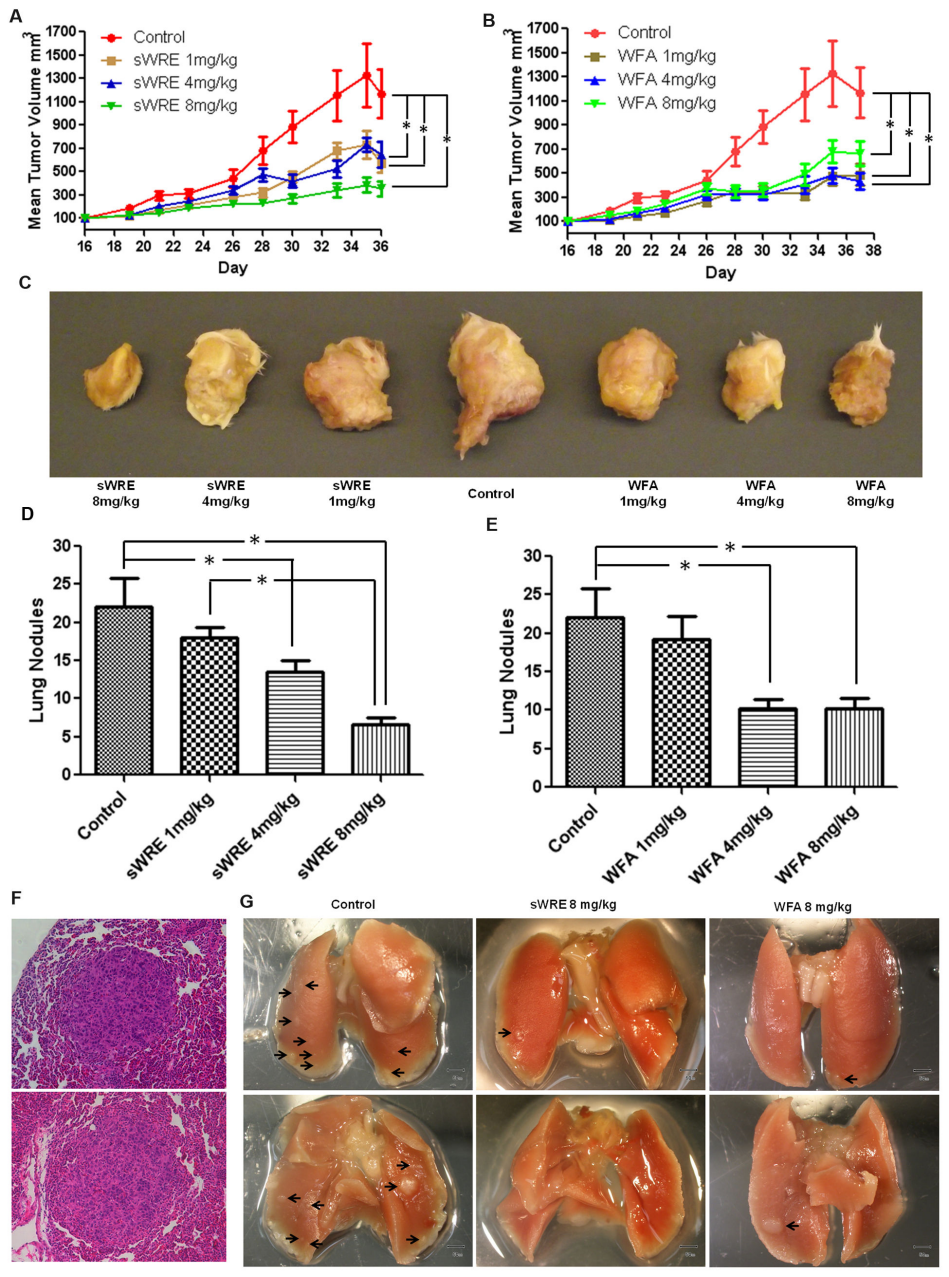
Anti-metastatic activity of sWRE and Withaferin A in an allograft breast cancer mouse model. (A and B) Mean tumor volume in mice treated with 1, 4, 8mg/kg of (A) sWRE or (B) Withaferin A (WFA) (* p<0.05 compared to control). (C) Representative images of the primary tumor in sWRE or WFA treated mice. (D and E) Bar graph showing the mean number of metastatic lung nodules in (D) sWRE or (E) WFA-treated mice (* p<0.05 compared to control). (F) Representative H&E staining images showing the histology of metastatic nodules in mouse lung; two examples shown. (G) Representative images of lung metastatic nodules (black arrows) in mice treated with vehicle control (9% EtOH) or sWRE or WFA at 8mg/kg.

To determine the anti-metastatic efficacy of sWRE and compare it to Withaferin A in a mouse model, the mouse mammary carcinoma 4T1 metastatic model was used. This model develops metastatic lesions in the lung, liver, and spleen 4-6 weeks post-injection of cells into the mammary fat pad. Mice were divided randomly into 4 groups with 10 mice in each group, and sWRE was given by oral gavage and Withaferin A by i.p. injection at 1, 4, and 8 mg/kg 3 times per week for 4 weeks. The dose range was selected based on the previous sWRE toxicity experiment in the BALB/c mouse (Figure 5). At all doses, primary tumor volume was decreased after 36 days of treatment with sWRE or Withaferin A (Figure 6A, B). Representative gross specimens of primary tumors show that both sWRE and Withaferin A treated tumors were smaller (Figure 6C). Importantly, the number of metastatic lung nodules significantly decreased in both 4 and 8 mg/kg groups in sWRE and Withaferin A treated mice (Figure 6D, E, and examples in Figure 6G). H&E staining confirmed the presence of micro-metastatic lesions in the lung (Figure 6F).

**Figure 7:**
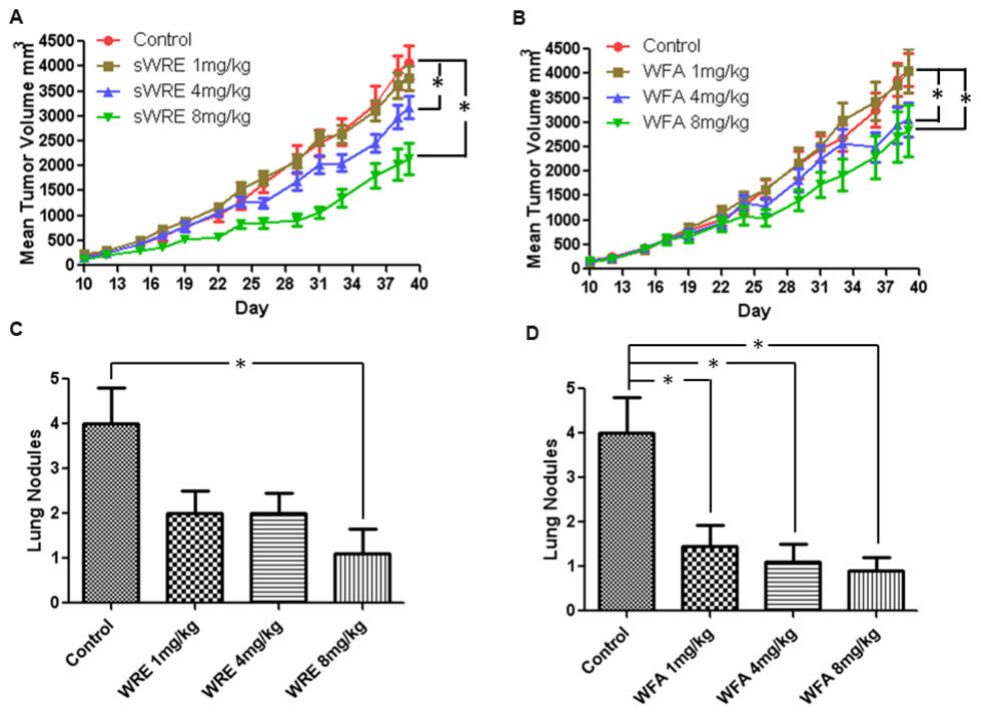
Anti-metastatic activity of sWRE and Withaferin A in an MDA-MB-231 xenograft breast cancer model. (A and B) Mean tumor volume in mice treated with 1, 4, 8mg/kg of (A) sWRE or (B) WFA (*, p<0.05 compared to vehicle control). Bar graph showing the mean number of metastatic lung nodules in (C) sWRE or (D) WFA-treated mice compared to vehicle control (9% EtOH; * indicated p<0.05 compared to control).

To further test the anti-metastatic efficacy of sWRE, a similar experiment using a xenograft model with human metastatic breast cancer MDA-MB-231 cells was performed. Cells were injected subcutaneously into the mammary fat pad of female athymic nude mice. Mice were administrated sWRE by oral gavage and Withaferin A by i.p. injection at concentration of 1, 4, and 8mg/kg 3 times a week for 4 weeks. Primary tumor volume was inhibited by sWRE at 4 and 8mg/kg doses. Similarly Withaferin A treatment also resulted in a reduction in tumor volume at 4 and 8mg/kg dose (Figure 7A, B). Importantly, both sWRE and Withaferin A showed similar efficacy and decreased the number of metastatic nodules in the lung at 8 mg/kg (Figure 7C, D).

## Discussion

Our data show that sWRE has similar anti-metastatic efficacy to pure Withaferin A in two *in vivo* mouse models (Figures 6 and 7). Specifically, sWRE significantly decreased metastatic lung nodule formation in the 4T1 model when given orally at 4 or 8 mg/kg and at 8 mg/kg in the MDA-MB-231 xenograft model, where in general sWRE had a more graded, dose-dependent effect on metastatic lung nodule formation. These results are similar to those observed with pure Withaferin A, which inhibited metastatic lung nodules at 4 and 8 mg/kg in both models (Figures 6 and 7). Furthermore, sWRE had nearly no toxicity based upon pathological analysis and monitoring of mouse weight at 8 mg/kg (Figure 5), which is similar to the previous observation that pure Withaferin A given i.p. also had minimal toxicity at a similar concentration [35]. Therefore, based upon these *in vivo* mouse studies we conclude that oral administration of sWRE has similar anti-metastatic efficacy as pure Withaferin A. Furthermore, these data would suggest that oral administration of WRE capsules that contain active Withaferin A could retain anti-metastatic efficacy in a clinical setting.

sWRE also inhibits cell motility and invasion *in vitro* at 0.5µM (Figure 3), which is well below its cytotoxic 24hr IC_50_ of 8µM or higher depending on the cell line (Figure 2). These data suggest that its ability to inhibit cell motility is distinct from its anti-proliferative activity, and thus may not be due to general cytotoxicity. We propose that inhibition of motility, could occur via vimentin inhibition, since we observed a prominent disruption of vimentin morphology in cells treated at 0.5µM and 1µM sWRE. It was previously shown that Withaferin A can bind directly to vimentin [28] and also disrupt vimentin morphology [29,30,35]; therefore, these results with sWRE are consistent with these previous studies showing vimentin inhibitory activity.

It is important to note that the role of vimentin in cell motility has remained controversial. Numerous reports show that vimentin is a classic EMT biomarker that is expressed in aggressive cell lines and tumors [46-53], and correlates with high grade cancer and metastatic disease [54-58]; Nevertheless, the precise molecular role of vimentin in cell motility remains largely undefined and there are several reports that induced vimentin expression in vimentin null cell lines does not impact motility [59,60]. Thus, it is still debatable as to why vimentin expression in certain contexts correlates with invasion (e.g., metastatic disease) while in other systems re-expression does not. Though Withaferin A and now sWRE are both shown to disrupt vimentin, we cannot directly rule out the possibility that both treatments inhibit metastasis through a vimentin-independent pathway.

sWRE can prevent EMT induction in the MCF10A EMT model (Figure 4) at 0.5µM, whereby sWRE treatment reverses vimentin and fibronectin induction and promotes E-cadherin expression. Furthermore, sWRE also potently inhibits TGFβ induced 3-D MCF10A spheroid invasion at both 0.1µM and 0.5µM (supplemental Movies). It remains unclear if the anti-EMT efficacy of sWRE is tied to its ability to inhibit vimentin, but one possibility is that vimentin inhibition by sWRE leads to its degradation and consequently a reversal of the EMT program. We did not observe changes in the vimentin transcript after sWRE treatment; therefore, we do not suspect that sWRE affects transcription of the EMT markers.

Though the major focus of these studies was on metastasis, it is interesting to note that higher concentrations of sWRE inhibited cell proliferation (Figure 2). Interestingly, the greatest anti-proliferative activity was observed in cell lines that were vimentin-positive suggesting a potential correlation between vimentin expression and cytotoxicity. Though vimentin is primarily linked to cell motility, there are reports that it functions in proliferation [61-64] and perhaps that is responsible for the observed cytotoxicity.

In triple negative breast cancers (estrogen, progesterone, and HER-2 negative), vimentin expression is correlated with poor prognosis as well as an aggressive and metastatic phenotype [64-68]. We observed that two of the three triple negative cell lines (MDA-MB-231 and Hs578-T) express vimentin. These cell lines were also the most sensitive to sWRE in cytotoxicity assays and sWRE inhibited metastasis in the MDA-MB-231 xenograft model. Though this data set is correlative and we cannot directly attribute the sensitivity to vimentin expression, we believe that these efficacy data suggest that sWRE has the potential to be used as an anti-metastatic in vimentin-positive tumors. Additional pharmacokinetic and pharmcodynamic data with sWRE will likely prove to be useful and will be the focus of future work.

## Supporting Information

Figure S1 (A) Wounding assay in MDA-MB 231 cells using low concentrations of sWRE (B) Western blotting of vimentin in MDA-MB 231 cells and BEAS-2B lung epithelial cells (C) MTT anti-proliferative assay in BEAS-2B cells with sWRE(D) Wounding assay in BEAS-2B lung epithelial cells using a range of sWRE concentrations.
(PDF)

Figure S2 (A) BCA assay quantifying protein concentration per cell in MDA-MB 231 cells after treatment at different timepoints with sWRE (*p<0.05).To do this, the total protein was divided by the number of cells in the well after treatment. (B) BCA assay quantifying protein levels per well after treatment at different timepoints with sWRE (*p<0.05).
(PDF)

Movie S1 Live cell imaging of MCF10A spheroids embedded in Matrigel and treated with TGF-beta.Time is in hours.
(AVI)

Movie S2 Live cell imaging of MCF10A spheroids embedded in Matrigel and treated with TGF-beta and sWRE at 100 nM.Time is in hours.
(AVI)
