# Supplementary figures and images for: Withania somnifera Root Extract Inhibits Mammary Cancer Metastasis and Epithelial to Mesenchymal Transition
Source: PLoS One. 2013 Sep 12;8(9):e75069. doi: 10.1371/journal.pone.0075069 (PMC3771884; doi:10.1371/journal.pone.0075069)

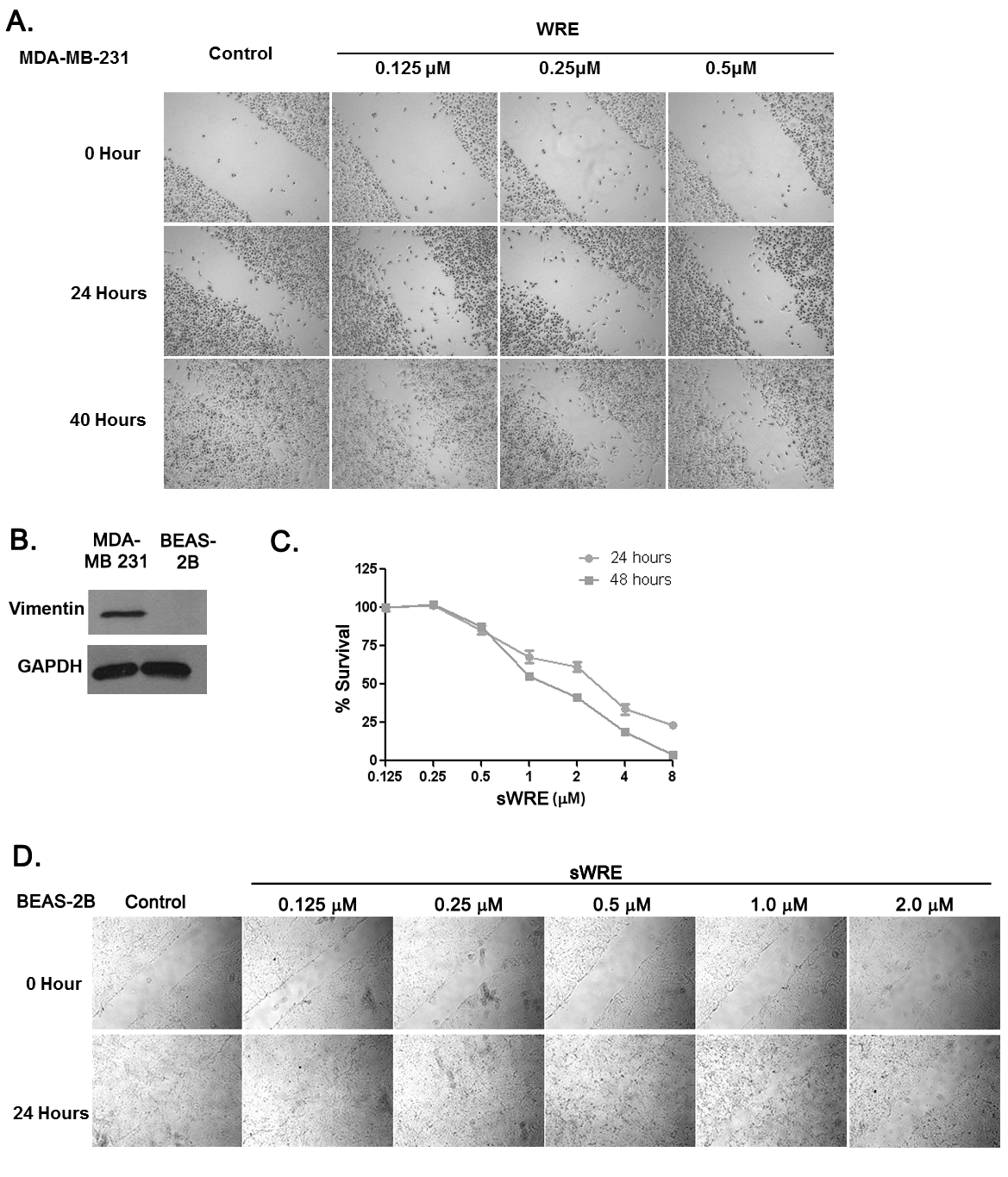

Supplement: Figure S1 — (A) Wounding assay in MDA-MB 231 cells using low concentrations of sWRE (B) Western blotting of vimentin in MDA-MB 231 cells and BEAS-2B lung epithelial cells (C) MTT anti-proliferative assay in BEAS-2B cells with sWRE(D) Wounding assay in BEAS-2B lung epithelial cells using a range of sWRE concentrations. (PDF) [file pone.0075069.s001.pdf]

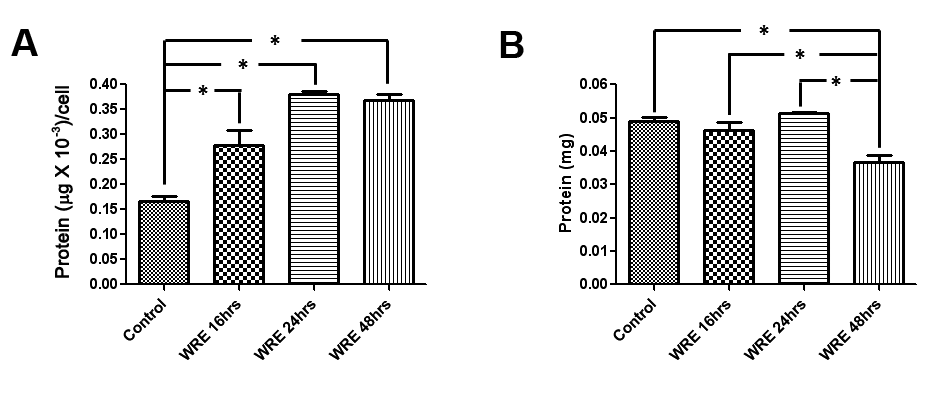

Supplement: Figure S2 — (A) BCA assay quantifying protein concentration per cell in MDA-MB 231 cells after treatment at different timepoints with sWRE (*p<0.05).To do this, the total protein was divided by the number of cells in the well after treatment. (B) BCA assay quantifying protein levels per well after treatment at different timepoints with sWRE (*p<0.05). (PDF) [file pone.0075069.s002.pdf]
